# Comparison of Cardiorenal Syndrome and Heart Failure: A Preliminary Study of Clinical, Cognitive, and Emotional Aspects

**DOI:** 10.3390/neurosci6040129

**Published:** 2025-12-15

**Authors:** Maria Pagano, Anna Anselmo, Giuseppe Micali, Fabio Mauro Giambò, Francesco Speciale, Daniela Costanzo, Piercataldo D’Aleo, Antonio Duca, Alessia Bramanti, Marina Garofano, Placido Bramanti, Francesco Corallo, Irene Cappadona

**Affiliations:** 1 IRCCS Centro Neurolesi Bonino-Pulejo, Via Palermo, S.S. 113, C.da Casazza, 98124 Messina, Italy; maria.pagano@irccsme.it (M.P.); antonio.duca@irccsme.it (A.D.);; 2 Department of Medicine, Surgery and Dentistry, University of Salerno, 84081 Baronissi, Italymgarofano@unisa.it (M.G.); 3 Faculty of Psychology, Università degli Studi eCampus, Via Isimbardi 10, 22060 Novedrate, Italy

**Keywords:** cardiorenal syndrome, heart failure, kidney failure, cognitive functioning, anxiety, depression

## Abstract

Background: Cardiovascular diseases (CVD) affect the heart and blood vessels. Cardiorenal syndrome (CRS) highlights the interaction between the heart and kidneys, worsening the clinical course. Assessing renal function is essential for risk stratification and guiding therapeutic decisions. Furthermore, cognitive and psychological aspects are often impaired in these patients. Aim: To compare clinical, cognitive, emotional, and quality of life parameters between patients with CRS and those with heart failure (HF) alone, and to assess the agreement between estimated glomerular filtration rate equations (Cockcroft–Gault and CKD-EPI). Methods: This observational study was conducted at the Cardiology Unit of the IRCCS Centro Neurolesi Bonino Pulejo “Piemonte” Hospital (Messina, Italy) between June 2024 and March 2025. Thirty participants aged 45–85 years were enrolled: 15 with type 1 cardiorenal syndrome (CRS group) and 15 with heart failure without cardiorenal syndrome (HF group). All participants had a confirmed diagnosis and provided informed consent. Clinical evaluation and standardized tests (MoCA, BDI-II, BAI, and SF-12v2) were administered. Statistical analyses were performed using *t*-tests, chi-square tests, and Bland–Altman analysis, with significance set at *p* < 0.05. Results: The two groups were comparable in body mass index and left ventricular ejection fraction. CRS patients had significantly higher serum creatinine and lower GFR with both equations. The two GFR equations were strongly correlated (r = 0.94; *p* < 0.0001). Bland–Altman analysis showed a mean difference of 5.80 mL/min (95% limits of agreement: –12.4 to +24.0 mL/min), indicating wide individual variability. No significant differences were found in cognitive performance or quality of life. However, CRS patients exhibited significantly higher depressive symptoms (BDI-II mean 11.33 ± 8.19 vs. 5.40 ± 6.68; *p* = 0.0384) and a trend toward higher anxiety (BAI mean 8.13 ± 4.73 vs. 4.67 ± 5.79; *p* = 0.0834). Conclusions: A multidisciplinary approach, including psychological support, is necessary for patients with CRS.

## 1. Introduction

**Figure 1:**
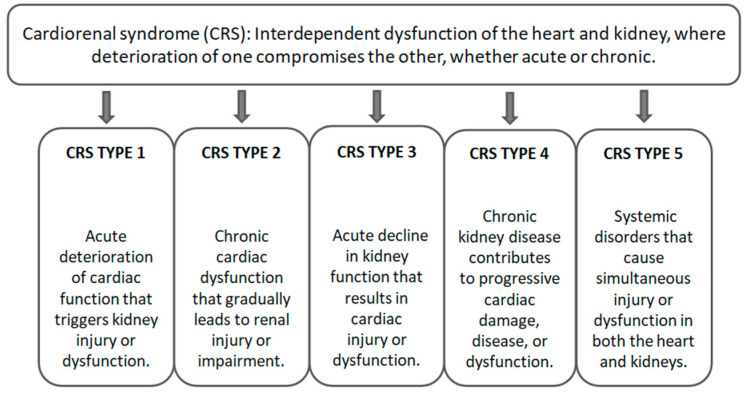
Classification of CRS by Acute Dialysis Quality Initiative (ADQI).

Cardiovascular disease (CVD) is one of the greatest public health problems worldwide, being one of the leading causes of morbidity, disability, and mortality [1]. The term CVD embraces a large range of pathological conditions affecting the heart and blood vessels, such as ischemic heart disease, cerebrovascular disease, rheumatic heart disease, heart failure, arrhythmias, and vascular malformations [2]. The presence of comorbidities such as diabetes mellitus, hypertension, obesity, and chronic kidney disease (CKD) significantly worsens the clinical course of CVD, increasing its severity and the likelihood of major cardiovascular events [3]. Heart failure (HF) represents the final clinical stage of many cardiovascular diseases and is characterized by the heart’s inability to maintain an adequate blood flow to meet the metabolic demands of body tissues [1]. It involves hemodynamic, neuroendocrine, and inflammatory alterations that lead to venous congestion, systemic hypoperfusion, and symptoms such as dyspnea, fatigue, and reduced exercise tolerance. Chronic HF, in particular, is associated with a high risk of exacerbations and recurrent hospitalizations, as well as a progressive deterioration in quality of life and survival [3]. This is the context in which cardiorenal syndrome (CRS) arises, a pathological condition that reflects the complex and reciprocal interactions between the heart and kidneys [4]. The term “cardiorenal syndrome” was introduced as early as the 1940s to describe this interdependence between the two organs [5]. The pathophysiology of CRS actively involves the activation of the sympathetic nervous system and the renin–angiotensin–aldosterone axis, together with inflammatory mechanisms, oxidative stress, and hemodynamic dysregulation [6]. These processes create a vicious cycle in which the dysfunction of one organ progressively compromises the other [7]. For descriptive purposes, the Acute Dialysis Quality Initiative (ADQI) proposed a classification of CRS [8] into five types based on clinical variants (Figure 1).

The incidence of CRS varies according to the clinical context, but occurs in up to 40% of patients hospitalized for heart failure and in 60% of patients with chronic kidney disease [9]. The clinical implications are severe, as cardiovascular disease mortality is the leading cause of death among patients with CKD in various regions of the world (Australia, Asia, Europe, and North America). Nearly 75% of patients with end-stage renal disease have left ventricular hypertrophy, while 40% have coronary artery disease. Conversely, over 60% of patients with heart failure have at least mild renal impairment, and about 20% have moderate to severe renal impairment [10].

In this context, assessing kidney function is very important when dealing with patients with CRS. The main predictive indicators are based on the glomerular filtration rate (GFR) including the two most commonly used formulas for estimating GFR: (i) the Cockcroft–Gault equation, which estimates creatinine clearance taking into account age, weight, sex, and serum creatinine; (ii) CKD-EPI, which estimates GFR based on age, sex, and creatinine [11]. Both formulas are clinically relevant when the estimated value is less than 60 mL/min, a threshold associated with a high risk of CKD progression and worsening cardiovascular prognosis. These parameters are not only essential for monitoring the progression of kidney disease, but also for stratifying cardiovascular risk and personalizing drug therapy in the presence of multimorbidity, as is frequently the case in patients with CRS [12].

Multimorbidity, defined as the coexistence of two or more chronic conditions, is increasingly common, especially among older adults. It compromises various aspects of daily life, including physical functioning, psychological well-being, treatment adherence, and personal autonomy [13]. Several studies show that quality of life (QoL) is significantly reduced in patients with multimorbidity, with further deterioration in complex cases such as cardiorenal syndrome. The coexistence of multiple organ dysfunctions requires greater attention in clinical management and multidisciplinary support [14,15,16].

In addition, anxiety and depression symptoms are not negligible, occurring in patients with multimorbidity with a prevalence that can reach 60% [17]. In patients with CRS, the psychological impact can be even more significant. Indeed anxiety and depression represent additional negative prognostic factors, associated with higher mortality and worse clinical outcomes [18,19]. An additional factor to consider is cognitive impairment, which is increasingly recognized as an integral part of multimorbidity in chronic patients [20]. In individuals with CRS, cognitive decline can manifest as attention and memory deficits, difficulties with planning and execution, and reduced decision-making abilities [21]. The causes are multifactorial, including reduced oxygen and nutrient supply to the brain, accumulation of uremic toxins, chronic systemic inflammation, and alterations in neuroendocrine metabolism [22,23]. Cognitive decline, in addition to compromising patient autonomy, could hinder the course of the disease in terms of doctor-patient communication, treatment adherence, and access to appropriate care [24].

However, despite growing evidence linking cardiorenal dysfunction to neuropsychological decline, there is a notable gap in the scientific literature regarding direct comparisons between patients with CRS and patients with isolated heart failure with similar clinical characteristics.

It is essential to fill this gap in order to clarify whether CRS itself entails an additional cognitive or emotional burden beyond that observed in heart failure alone. In the clinical sample recruited at the Cardiology Unit of the IRCCS Centro Neurolesi Bonino Pulejo, a prevalence of patients with cardiorenal syndrome and those with isolated heart failure was observed. These two conditions share a common cardiovascular background but differ in the presence of renal dysfunction. This observation provided the rationale for a comparative analysis between the two groups, aimed at exploring the impact of additional renal impairment on neuropsychological aspects and overall patient well-being. Such an approach allows for a deeper exploration of the heart–kidney–brain relationship in a real-world clinical context, contributing to a more integrated understanding of the interactions between renal function, emotional state, and cognition in chronic patients, which has already been studied in complex syndromes [25,26].

In this background, this study aims to investigate differences in clinical, cognitive, emotional, and quality of life parameters between patients with cardiorenal syndrome (CRS) and those with heart failure (HF) alone. In addition, the concordance between the Cockcroft–Gault and the CKD-EPI equations will be evaluated.

## 2. Materials and Methods

This observational study aims to investigate any differences in terms of cognitive impairment, anxiety, and depression between patients with cardiovascular disease, with or without cardiorenal syndrome. Participants were recruited from among patients attending the Cardiology Unit of the IRCCS Centro Neurolesi Bonino Pulejo P.O. “Piemonte” in Messina as part of the observational clinical trial entitled “Prevention and Treatment of Cardiovascular Disease: Protocol for the Assessment of Psychological, Neuropsychological Implications and Associated Disorders in Cardiovascular Patients” [27], approved by the local ethics committee of the IRCCS Centro Neurolesi Bonino Pulejo Neurolesi (Register of opinions: 8/2024) and registered on ClinicalTirals.gov (ID: NCT06413823). All subjects provided informed consent.

### 2.1. Population

**Figure 2:**
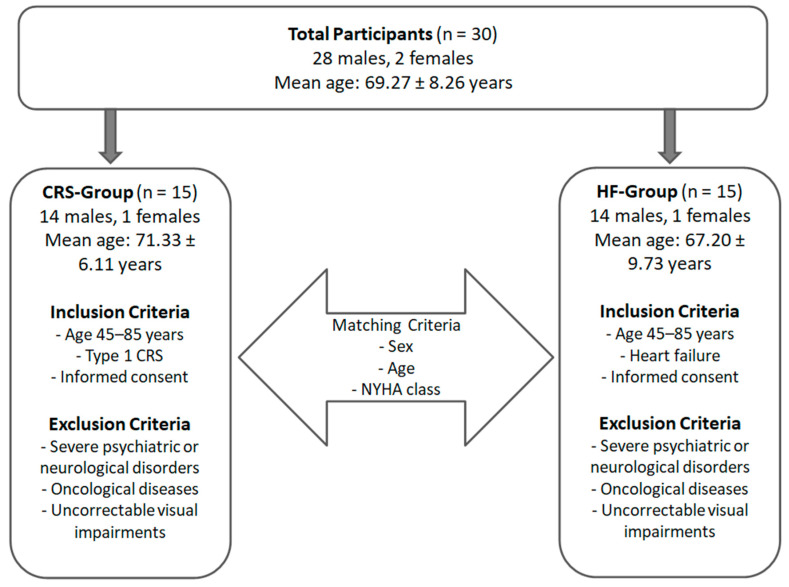
Sample Distribution.

This study included a total of 30 participants (28 males, 2 females; mean age: 69.27 ± 8.26 years), divided into two groups (Figure 2).

The first group included 15 participants living with cardiorenal syndrome (CRS-Group) while the second group included 15 participants living with only heart failure (HF-Group). The HF-Group was selected, matching subjects by sex, age New York Heart Association (NYHA) functional class to ensure clinical comparability between the groups. Inclusion and exclusion criteria was summarized in Table 1.

### 2.2. Clinical Measures

For each patient, the following clinical parameters were extracted from medical records such as body mass index (BMI), left ventricular ejection fraction (LVEF), New York Heart Association (NYHA) functional class, and serum creatinine levels. Additionally, the glomerular filtration rate (GFR) was estimated for all participants using both the Cockcroft–Gault formula and the CKD-EPI (Chronic Kidney Disease Epidemiology Collaboration) equation.

Body mass index (BMI) is a widely used screening tool that assesses an individual’s body weight in relation to their height, providing a general indication of nutritional status. It is calculated by dividing a person’s weight in kilograms by the square of their height in meters (kg/m^2^). According to the World Health Organization (WHO) international classification, a BMI below 18.5 is considered underweight, while a BMI between 18.5 and 24.9 is considered normal or healthy weight. Values between 25.0 and 29.9 are classified as overweight, while a BMI of 30.0 or above is classified as obese. Obesity is further divided into three classes: (i) Class I (BMI 30.0–34.9); (ii) Class II (BMI 35.0–39.9); (iii) Class III (BMI > 40) [28].

Left ventricular ejection fraction (LVEF) plays a central role in the diagnosis, classification, and therapeutic management of heart failure (HF), and it also serves as a key inclusion criterion in clinical trials on HF. According to current European Society of Cardiology (ESC) guidelines, heart failure is categorized based on LVEF into three groups: HF with preserved ejection fraction (HFpEF) when LVEF is ≥50%, HF with mildly reduced ejection fraction (HFmrEF) when LVEF ranges between 40 and 49%, and HF with reduced ejection fraction (HFrEF) when LVEF is <40% [29].

The NYHA functional class is a clinical classification system developed by the New York Heart Association to assess the severity of symptoms in patients with heart failure. It is divided into 4 classes: (i) Class I indicates no symptoms during ordinary activity; (ii) Class II indicates mild symptoms during ordinary activity; (iii) Class III indicates marked limitation of physical activity; (iv) Class IV indicates the presence of symptoms at rest [30].

Serum creatinine is a metabolic waste product filtered by the kidneys. Elevated serum creatinine levels typically indicate impaired kidney function. It is commonly used as a surrogate marker in estimating kidney function [31].

The Cockcroft–Gault (CG) formula estimates creatinine clearance, which serves as a proxy for GFR. It takes into account age, body weight, sex, and serum creatinine and is particularly useful for drug dosing in patients with renal impairment [32].

The Chronic Kidney Disease Epidemiology Collaboration (CKD-EPI) equation [33] is a most recent method for estimating GFR, especially at higher levels of renal function. It takes into account age, sex, and serum creatinine. Both the CG and CKD-EPI equations are recommended for staging chronic kidney disease (CKD) by calculating estimated GFR (eGFR) when associated by kidney damage.

### 2.3. Neuropsychological Measures

All participants were evaluated using a standardized battery of psychometric instruments, including the Montreal Cognitive Assessment (MoCA), the Beck Anxiety Inventory (BAI), the Beck Depression Inventory-II (BDI-II) and the Short Form Health Survey version 2 (SF-12v2).

The MoCA is a brief cognitive screening tool widely used in both clinical and research settings for the detection of mild cognitive impairment (MCI). It assesses six cognitive domains: visuospatial/executive functions, attention, language, memory (delayed recall), and orientation. A score of less than 26 out of 30 is generally interpreted as suggestive of possible cognitive impairment [34].

The BAI is a 21-item self-administered inventory aimed at evaluating the severity of anxiety symptoms experienced during the previous week. Each item is rated from 0 (not present) to 3 (severely present), with total scores derived by summing the individual item ratings [35].

The BDI-II is a 21-item self-report questionnaire designed to assess the intensity of depressive symptoms over the preceding two weeks. Items are rated on a 4-point scale ranging from 0 (no symptoms) to 3 (severe symptoms), with total scores ranging from 0 to 63. Higher scores reflect greater severity of depressive symptomatology [36].

The SF-12v2 is a validated self-report questionnaire used to assess health-related quality of life in physical and mental domains. Comprising 12 items from the longer SF-36, it offers a reliable summary of overall functional health status. Scores are standardized to a mean of 50 and a standard deviation of 10, based on the general population. Higher scores indicate better perceived health while lower scores reflect greater impairment [37].

All instruments were administered in the validated Italian version, which has good psychometric properties of reliability and validity [34,35,36,37].

### 2.4. Statistical Analysis

Statistical analyses were conducted to assess differences between the CRS and HF groups. The distribution of the sample was assessed using independent sample *t*-tests for continuous variables and Pearson’s chi-square test for categorical variables. Continuous variables, including age, serum creatinine, estimated glomerular filtration rate (both the Cockcroft–Gault and CKD-EPI equations), ejection fraction, cognitive scores, affective symptoms, and quality of life indicators, were analyzed using independent sample *t*-tests, as all variables were continuous and approximately normally distributed. In addition, to assess the agreement between the Cockcroft–Gault and CKD-EPI equations in estimating GFR, a Bland–Altman analysis was performed to calculate the mean difference between the two methods and define the limits of agreement, and a Pearson correlation analysis was used to assess the strength and direction of the linear association between CG and CKD-EPI estimates. Descriptive statistics, including means and standard deviations, were calculated for each variable within both groups. A significance threshold of α = 0.05 was adopted for all comparisons.

## 3. Results

### 3.1. Sociodemographic Characteristics of the Sample

The sociodemographic characteristics of the sample are shown in Table 2.

### 3.2. Clinical Measures Comparison

Comparison of clinical parameters between the two groups revealed that BMI and LVEF did not differ significantly. This indicates that both cohorts were well matched in terms of cardiac function and overall body composition. In contrast, serum creatinine levels were significantly higher in the CRS group, confirming the presence of renal dysfunction. Consequently, glomerular filtration rate (GFR), calculated using both the Cockcroft–Gault and CKD-EPI formulas, was significantly lower in the CRS group than in the HF group. This results are summarized in Table 3.

### 3.3. Equations Comparison

The comparison between the Cockcroft–Gault and EPI-KCD formulas for estimating glomerular filtration rate showed a very strong correlation, as demonstrated by Pearson’s test (r = 0.94; *p* < 0.0001). The Bland–Altman analysis revealed an average difference between the two methods of 5.80 mL/min (*p* = 0.1373, *t*-test). The limits of agreement calculated using the same method are wide, ranging from −34.93 to +46.53 mL/min. These values indicate significant intra-individual variability. Indeed in some subjects, the difference between the two formulas exceeds 30 mL/min and this discrepancy could affect the clinical classification of renal function (Table 4).

### 3.4. Neuropsycological Measures Comparison

Neuropsychological assessment showed that cognitive function, measured using MoCA, did not differ significantly between the CRS group and the HF group, suggesting comparable cognitive performance in both populations. Depressive symptoms assessed using BDI-II were significantly more pronounced in the CRS group, indicating a higher emotional burden in patients suffering from both cardiac and renal dysfunction. Anxiety levels measured by BAI were also tendentially higher in the CRS group, although this difference did not reach statistical significance. Quality of life, assessed by the SF-12v2 questionnaire based on mental, physical, and total components, was not significantly different between groups. Neuropsycological measures comparison results are summarized in Table 5.

## 4. Discussion

The distribution of the sample by gender, NYHA class, and age showed that the clinical parameters between the two groups were homogeneous. This was confirmed by the absence of significant differences in left ventricular ejection fraction (LVEF) and the expected presence of significant differences in serum creatinine and eGFR estimated using the CG and CKD-EPI equations. Although the CG and CKD-EPI equations provide reasonably accurate estimates, they are not overlayable. The discrepancy between the two formulas was noted in a previous study [38], which recognized the greater accuracy of CKD-EPI. In fact, although the Cockcroft–Gault equation is still widely used, particularly in drug reconciliation, the CKD-EPI formula is considered the most accurate and recommended for the clinical assessment of renal function in clinical practice [39].

This finding reinforces the clinical relevance of selecting appropriate GFR estimation methods in multimorbid patients, where small differences in renal indices can have significant implications for drug dosing and risk stratification.

The lack of significance in the BMI comparison, which showed an average overweight situation for both groups, confirms the importance of lifestyle in chronic diseases, including cardiovascular and renal diseases [40]. Factors such as an unbalanced diet, a sedentary lifestyle, and being overweight are among the risk factors for the development of chronic diseases [41]. In addition, it is common for a vicious cycle to develop in which chronic diseases, once fully developed, can lead to reduced physical capacity to perform activities, increasing sedentary lifestyles and aggravating the diseases, especially in cases of multimorbidity [42]. This can have consequences on quality of life. In fact, the quality of life assessed in this study using the SF-12v2 questionnaire showed no significant differences between the groups. This may reflect a generally reduced quality of life in both populations due to the burden of chronic heart failure, regardless of renal function status [43]. Alternatively, this apparent similarity may indicate a floor effect, in which quality of life is already substantially compromised by heart failure itself, leaving little room for additional deterioration attributable to renal dysfunction. In contrast to our findings, a recent study states that chronic kidney disease leads to a reduction in quality of life [44] and a systematic review and meta-analysis states that quality of life progressively decreases with increasing multimorbidity [13]. The discrepancy with previous evidence highlights the importance of contextual factors such as disease stage, treatment optimization, and psychosocial support, which may modulate the perceived quality of life independently of renal function indices.

Neuropsychological assessments provided further information on the cognitive and emotional status of the two groups. Cognitive performance, measured using the MoCA test, did not differ significantly between CRS and HF patients, but this result should not be interpreted as evidence of the absence of cognitive impairment related to renal dysfunction. Rather, it indicates that within this sample, there was no statistically significant difference in MoCA scores, which may reflect limited statistical power due to the relatively small sample size. Hence, a Type II error (false negative) cannot be excluded, and larger samples or more sensitive cognitive measures might detect subtle deficits.

Depressive symptoms, assessed using the BDI-II, were significantly more pronounced in the CRS group. This finding highlights a greater emotional burden associated with the combined presence of cardiac and renal dysfunction. This association, however, should be interpreted cautiously given the cross-sectional design, which precludes causal inference. It remains unclear whether depression is a consequence of CRS-related physiological and psychosocial stressors, or whether pre-existing depressive states may exacerbate disease progression through behavioral and neuroendocrine mechanisms [45].

Anxiety levels, measured using the BAI, also tended to be higher in the CRS group, although the difference did not reach statistical significance. The trend observed, consistent with prior evidence [46], suggests that emotional dysregulation may be an early marker of distress in CRS, warranting further longitudinal investigation. A recent observational study and systematic review highlighted a varied prevalence of cognitive decline, anxiety, and depression in people with cardiovascular disease, emphasizing the importance of multidimensional assessment to improve clinical management [47]. The interaction between kidney disease, cognitive decline, and depression has also been the subject of recent studies [48]. Several authors have already suggested the need for a holistic and multidisciplinary approach in the treatment of cardiorenal syndrome; however, many programs do not include treatment for the emotional effects that may be related to these conditions [49,50,51].

Our findings further support this view by underscoring that even in the absence of overt cognitive impairment, emotional symptoms may represent an underrecognized therapeutic target in CRS management. Future research should integrate psychological and behavioral interventions into cardiorenal care pathways, as proposed by recent problem-oriented frameworks [52,53].

### Strengths and Limitations

This study has several strengths, including the use of well-matched comparison groups and the inclusion of neuropsychological assessments that provide a more comprehensive clinical picture by exploring cognitive and emotional health. However, it is not without limitations. First, there is the relatively small sample size, which may reduce the statistical power of some comparisons. Indeed, the study involved a total of 30 participants, divided equally into two groups, which further reduced the number of individuals in each sample, confirming the need for larger studies to validate these findings. Furthermore, from a demographic point of view, women are underrepresented. Although policies and initiatives have been implemented to improve adherence and reduce disparities in access and dropout rates for rehabilitation services [52,54,55], gender imbalance still persist in this context [56]. As a result, the capacity to perform robust subgroup analyses or reach firm conclusions about gender-specific differences is limited. Therefore, the results should be interpreted with caution, and further research with larger and more balanced samples is needed to confirm and expand on these findings. The cross-sectional design prevents causal inferences and limits understanding of disease progression. Furthermore, potential confounding variables, such as medications or additional comorbidities such as diabetes, were not fully controlled for. In particular, data regarding prevalent conditions such as diabetes, hypertension, and dyslipidemia were not comprehensively collected or analyzed. As these factors are common in both CRS and HF populations and are known to influence renal and neuropsychological function, their unmeasured effects may have influenced the observed results. Therefore, the study population may not be representative of broader clinical settings and the findings should be interpreted with caution, and future studies should include a more detailed assessment and control of these comorbidities to strengthen the validity of the results.

## 5. Conclusions

The study showed that, while presenting a similar distribution of baseline clinical and cardiac characteristics, including NYHA class, patients with cardiorenal syndrome exhibited significantly worse renal function and more pronounced depressive symptoms compared with patients with heart failure alone. The two GFR estimation formulas (Cockcroft–Gault and CKD-EPI) were strongly correlated, although some individual discrepancies were observed. No significant differences in cognitive function or quality of life were found between the groups. These findings support the need for a clinical approach that also takes psychological aspects into account in the management of patients with cardiorenal syndrome.

Future research should include large-scale longitudinal studies that include gold standard GFR measurements and consider additional biomarkers such as cystatin C. It is also important to emphasize that the results highlight the need for a holistic and multidisciplinary approach to patient care. This should integrate nephrology, cardiology, psychology, and mental health support, ensuring that emotional well-being is addressed alongside physical health.

## Figures and Tables

**Table 1:** Inclusion and exclusion criteria.

Criteria	CRS-Group	HF-Group
Inclusion Criteria	− Age between 45 and 85 years; − Confirmed diagnosis of type 1 cardiorenal syndrome − Provided informed consent	− Age between 45 and 85 years − Confirmed diagnosis of heart failure without cardiorenal syndrome − Provided informed consent
Exclusion Criteria	− Severe psychiatric or neurological disorders − Oncological diseases − Uncorrectable visual impairments	− Severe psychiatric or neurological disorders − Oncological diseases − Uncorrectable visual impairments
Matching Criteria	Matched with HF group by Sex, Age, NYHA functional class	Matched with CRS group by: Sex, Age, NYHA functional class

**Table 2:** Participants’ sociodemographic characteristics.

Variables	CRS-Group	HF-Group	*p*-Value
Gender (*n*)			
M	14	14	1.00
F	1	1	
Age mean (SD)	71.33 (6.11)	67.20 (9.73)	0.17
NYHA class (n)			
I	6	4	0.70
II	9	11	

**Table 3:** Clinical measure comparison results.

Variable	CRS Mean (SD)	HF Mean (SD)	*p*-Value
BMI (kg/m^2^)	27.43 (5.96)	27.43 (5.54)	0.99
LVEF (%)	51.60 (9.39)	54.27 (9.06)	0.44
Serum Creatinine (mg/dL)	2.45 (2.64)	0.90 (0.23)	0.03
GFR-Cockcroft–Gault (mL/min)	46.85 (22.78)	96.25 (35.36)	0.0001
GFR-CKD-EPI (mL/min/1.73 m^2^)	45.58 (26.50)	85.93 (16.28)	<0.0001

**Table 4:** Individual kidney function distribution compared via Cockcroft–Gault and CKD-EPI equations.

CRS-Group																
CKD Range (Value eGFR)	Equation Used	Subjects														
		1	2	3	4	5	6	7	8	9	10	11	12	13	14	15
Normal (eGFR ≥ 90)	Cockcroft–Gault															
	CKD-EPI															
Mild impairment (90 > eGFR ≥ 60)	Cockcroft–Gault		X										X	X	X	X
	CKD-EPI												X	X	X	X
Moderate impairment (60 > eGFR ≥ 30)	Cockcroft–Gault				X	X		X	X		X	X				
	CKD-EPI		X		X	X		X	X		X	X				
Severe impairment (30 > eGFR ≥ 15)	Cockcroft–Gault	X		X			X									
	CKD-EPI	X					X									
Kidney failure (eGFR < 15)	Cockcroft–Gault									X						
	CKD-EPI			X						X						
**HF-Group**																
**CKD Range (Value eGFR)**	**Equation Used**	**Subjects**														
		**1**	**2**	**3**	**4**	**5**	**6**	**7**	**8**	**9**	**10**	**11**	**12**	**13**	**14**	**15**
Normal (eGFR ≥ 90)	Cockcroft–Gault	X			X				X			X	X	X	X	X
	CKD-EPI			X		X			X			X	X	X	X	X
Mild impairment (90 > eGFR ≥ 60)	Cockcroft–Gault		X	X		X	X			X	X					
	CKD-EPI	X	X		X		X			X	X					
Moderate impairment (60 > eGFR ≥ 30)	Cockcroft–Gault							X								
	CKD-EPI							X								
Severe impairment (30 > eGFR ≥ 15)	Cockcroft–Gault															
	CKD-EPI															
Kidney failure (eGFR < 15)	Cockcroft–Gault															
	CKD-EPI															

**Table 5:** Neuropsycological measures comparison results.

Variable	CRS Mean (SD)	HF Mean (SD)	*p*-Value
MoCA	24.93 (3.41)	24.67 (2.55)	0.8103
BDI-II	11.33 (8.19)	5.40 (6.68)	0.0384
BAI	8.13 (4.73)	4.67 (5.79)	0.0834
SF-12 Mental Score (%)	66.42 (11.06)	67.65 (9.64)	0.7482
SF-12 Physical Score (%)	62.33 (5.63)	63.00 (10.14)	0.8255
SF-12 Total Score (%)	64.68 (6.57)	65.68 (6.59)	0.6811

## Data Availability

The data that support the findings of this study are not openly available due to reasons of sensitivity and are available from the corresponding author upon reasonable request.
